# Risk Factors for Injury in Border Collies Competing in Agility Competitions

**DOI:** 10.3390/ani14142081

**Published:** 2024-07-16

**Authors:** Arielle Pechette Markley, Abigail B. Shoben, Nina R. Kieves

**Affiliations:** 1 Department of Veterinary Clinical Sciences, College of Veterinary Medicine, The Ohio State University, Columbus, OH 43210, USA; kieves.1@osu.edu; 2 Red Sage Integrative Veterinary Partners, Fort Collins, CO 80524, USA; 3 Division of Biostatistics, College of Public Health, The Ohio State University, Columbus, OH 43210, USA; shoben.1@osu.edu

**Keywords:** Border Collie, canine agility, risk factors, injury, veterinary sports medicine

## Abstract

**Simple Summary:**

Injuries in agility dogs are common. Border Collies are the most common dog breed participating in agility and their reported injury rate is notably higher than that of other breeds. Understanding the risk factors for injury allows for improved education and awareness and the development of injury prevention strategies, as well as improved management plans post-injury. Our aim was to identify which demographic, training, and competition variables were associated with the injury risk specifically in Border Collies. An increased risk of injury in Border Collies was associated with the following factors: the height of jumps relative to the shoulder height, the number of competitions at the national level, the age at the beginning of elbow height jumping, and the number of weekends spent competing during the year. Other factors associated with the injury risk included the age at the beginning of backside jumps, the acquisition of the dog from a breeder, and the age of the handler. These data provide valuable information about the possible risk factors for injury in Border Collies and highlight the need for prospective studies on injuries in agility dogs and the relationships between injuries and the breed, conformation, and genetics.

**Abstract:**

Border Collies are the most common breed in agility and their reported injury rate is much higher than that of other breeds. We aimed to identify demographic, training, and competition variables associated with the injury risk for this breed. We hypothesized that higher jump heights and competition at national/international levels would increase the injury risk. Data were collected from an internet-based survey. A logistic regression model was built using backward selection. There were 934 Border Collies in the sample, with 488 reporting an injury. The jump height relative to the shoulder height was associated with injury, with dogs jumping noticeably above or below shoulder height more likely to report a history of injury. Other identified risk factors included the number of weekends spent competing/year, the number of competitions at the national level, the age when starting elbow height jumps and backside jumps, the acquisition of the dog from a breeder, and the age of the handler. Factors associated with prolonged injury (>3-month duration) were the age when starting elbow height jumps and having a veterinary assistant as a handler. Border Collies jumping above shoulder height had an increased risk of injury. However, those jumping below shoulder height were also at a higher risk, which could have been due to reverse causality. Similarly, the observed associations regarding differences based on the number of trial weekends/year may have been impacted by reverse causality as well. The increased risk of injury with elbow height jump training at <10 months of age may indicate that the repetitive impact of jump training prior to skeletal maturity negatively influences musculoskeletal development. These data provide valuable information for further prospective studies.

## 1. Introduction

Agility is a canine performance sport that demands speed, precision, and a high degree of athletic ability. The sport requires dogs to navigate a timed obstacle course containing jumps, tunnels, and other obstacles, in a pre-determined sequence, without errors (faults). Prior work suggests that there is a high lifetime injury rate among dogs competing in agility, ranging from 32% to 42% [1,2]. Previous studies have suggested a variety of potential risk factors for these injuries, including the Border Collie breed, competition at a higher level, an increased dog weight–height ratio, the handler’s experience, and the jump height [3,4,5,6].

The Border Collie breed is a popular breed for agility and retrospective surveys have indicated that Border Collies are the most common breed competing in agility, representing between 16% and 22% of the dogs in surveys completed by agility dog handlers [1,5,7]. These data are consistent with data from agility organizations as well. Of the 20,607 dogs with at least one qualifying agility run in American Kennel Club (AKC) events in 2022, 3190 (15.4%) were Border Collies [8].

Prior owner-reported survey studies have revealed that Border Collies are also injured at a higher rate than other breeds among dogs competing in agility [1,4,5,9]. The most common injuries in agility Border Collies include shoulder injuries, iliopsoas injuries, and digit injuries [1]. In particular, Border Collies are at a higher risk of developing iliopsoas injuries than other breeds [1,10]. Due to the high rate of injury within the breed, understanding the risk factors associated with injury development within Border Collies specifically, and how these risk factors compare with those in other breeds and agility dogs overall, may provide insight into both breed-related factors and modifiable training and competition risk factors. No previous studies have evaluated breed-specific risk factors in agility dogs.

Understanding the risk factors for injury allows for improved education and awareness and the development of injury prevention strategies, as well as improved management plans post-injury. Additionally, understanding these risk factors specifically among Border Collies enables researchers to study the factors associated with injury that may be confounded by the breed or dog size, which can be challenging to control for in statistical analyses. Our aim was to identify which demographic, training, and competition variables were associated with the injury risk specifically in Border Collies. We hypothesized that jumping at higher jump heights would be associated with an increased injury risk, based on previous biomechanical studies [11,12]. We also hypothesized that Border Collies with handlers who had competed at the national/international level would be associated with an increased injury risk, based on previous injury risk factor studies [4].

## 2. Materials and Methods

### 2.1. Survey Data

The specifics of the internet survey have been previously published [1,13]. Owners were asked in detail about injuries that kept their dogs from training or competing in agility for at least a week, as well as demographic variables, training history, and competition history. The survey was open to participants from around the world, but was available only in English. The dog had to have competed in agility at least within the past 3 years to be eligible, and owners were instructed to select randomly (based on name) if they had more than one eligible dog. The survey and research protocol were reviewed, approved, and determined exempt by the institutional review board at The Ohio State University (Study ID #2019E0892).

The present analysis was restricted to only Border Collies. The primary outcome was any history of previous injury keeping the dog from agility training or competition for at least a week. A secondary outcome was “prolonged” injury, which was defined as an injury that kept the dog from training or competition for more than three months. Potential risk factors were grouped into three blocks: demographic variables (e.g., dog age, weight, sex, handler agility experience, breed, country), competition-level variables (e.g., primary organization, frequency of competing, surfaces of competitions), and initial training factors (age at each obstacle training, trained contact obstacle behavior, method of weave training, etc.). With the exception of questions asking about past events (i.e., initial training), the questions asked about current practices when the dog was “healthy and actively training”.

### 2.2. Statistical Analysis

For the primary analysis, logistic regression was used to identify factors associated with a history of previous injury (yes/no). All models were adjusted for dog age in an effort to control for differences in exposure time. Categorical variables were considered using the multivariate Wald test of the null hypothesis that all categories had the same odds of injury. Similarly, height (continuous) and weight (continuous) were tested together using a multivariate Wald test that neither was associated with the outcome. This approach ensured that either both height and weight would be included or neither would be included, in an effort to characterize the impact of greater weight for the same height. A sensitivity analysis was conducted considering height alone as a covariate (without weight).

The final model was built using backward selection, such that all variables in the final model were significant at *p* < 0.05. Candidate variables were first considered within their blocks (i.e., demographic, competition, or training history). Within each block, variables that were promising (at *p* < 0.20) in age-adjusted only models were carried forward to a second stage where all variables that passed the initial screening step were subjected to a backward selection process until all variables remained promising for further study (at *p* < 0.20) [14]. A final stage took all variables that were promising from each block and conducted a final backward selection process until all variables in the final model were significant (at *p* < 0.05). The same process was used for the secondary prolonged injury outcome.

An exploratory analysis was also conducted to investigate differences in the types of injuries reported based on the height of the dog. Border Collies were grouped into three height classes based on the height at the withers (<19 inches, 19–21 inches, >21 inches), and the percentage of dogs in each group reporting an injury to a specific body part was calculated. Differences in the distribution of the reported injuries by height were examined with Chi-square tests among all types of injuries where more than 5% of the dogs reported an injury to that body part. As an exploratory analysis, we considered *p* < 0.05 to be significant, but we also report the results after a Holm correction for multiple comparisons.

All analyses were conducted using Stata v15.1.

## 3. Results

### 3.1. Sample

There were 934 Border Collies in the sample, representing 22% of the 4197 total dogs. Of the 934 Border Collies, 488 (52.3%) were reported to have at least one injury keeping them from training or competing for at least a week, and 195 (20.9%) reported at least one prolonged injury keeping them from training or competing in agility for more than three months. Among the non-Border Collies in this sample, these percentages were 38.3% and 13.3%, respectively. Among Border Collies reporting at least one injury (*n* = 488), approximately half (*n* = 250, 51.6%) reported more than one such injury. A similar percentage of more than one injury was observed among non-Border Collies reporting at least one injury (611 of 1246, 49.0%, Table 1).

### 3.2. Injury Risk Factors

**Figure 1:**
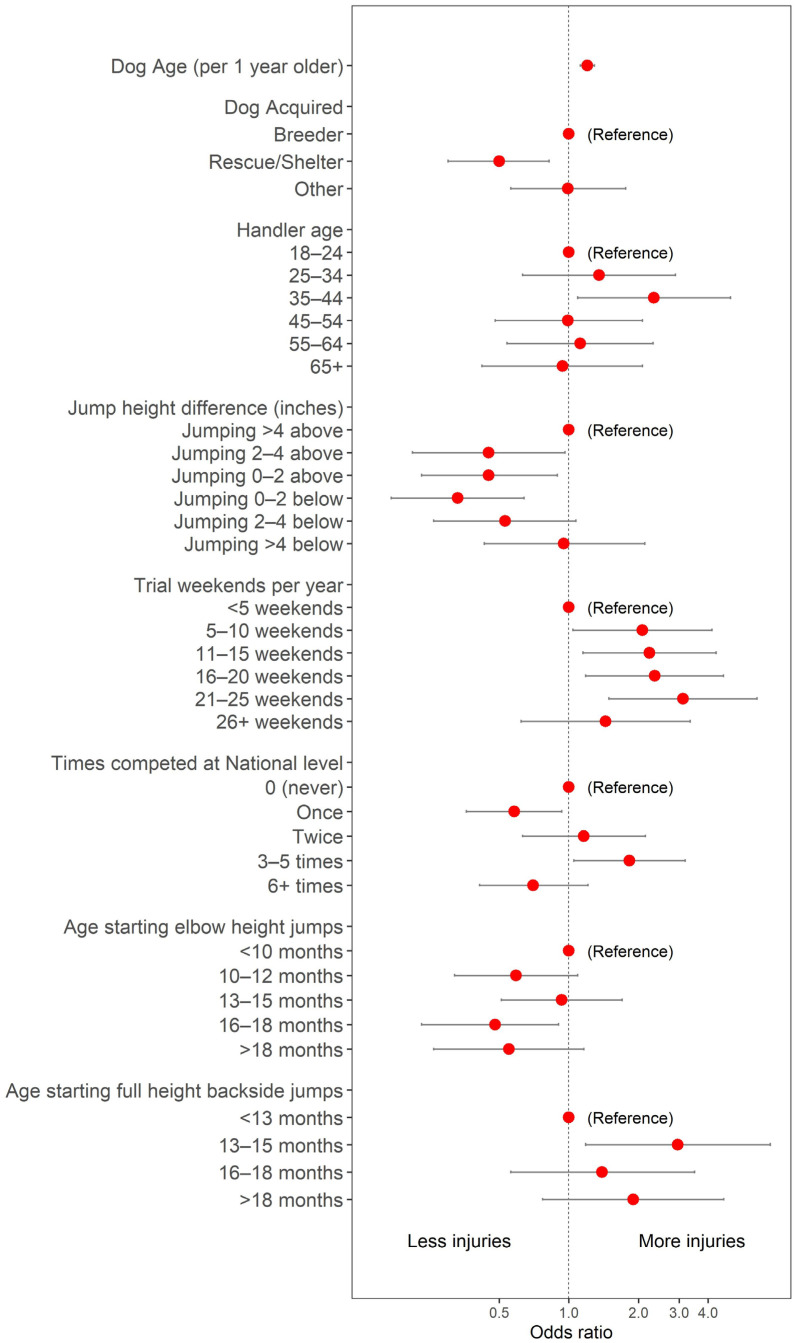
Coefficients from the final adjusted model of any injury history.

All variables considered, and whether or not they were retained in each stage of the model building process, are shown in Table 2. In the primary model of factors associated with any injury history, seven variables, in addition to the dog age, were found to be significant (Table 3, Figure 1). The jump height relative to shoulder height was significantly associated with injury, with dogs jumping noticeably above (>4” above) and noticeably below (>4” below) most likely to report any injury history. The number of weekends on which a dog typically competed in a year was also significantly associated with an injury history. Dogs competing on a small number of weekends (<5) were least likely to report an injury history, but dogs competing on most weekends (26 or more in the year) were the group with the second-lowest odds of reporting an injury history in the adjusted model. There was an observed qualitative trend towards increasing odds with an increasing number of weekends from 6–10 up to 21–25 weekends per year. The number of times that the dog had competed at a national competition was also associated with an injury history. The highest odds were observed for dogs who had competed three to five times at the national level, with the lowest odds observed among dogs who had competed exactly one or more than five times. The age at which dogs were reported to have started training with elbow height jumps was also associated with any injury history, with the highest odds observed among those who were reported to have started training at <10 months or between 13–15 months old and the lowest odds among those who started after 15 months (16–18 and >18 months). Similarly, the age at which a dog was reported to have started full height backside jumps was associated with an injury history. However, the lowest risk was observed among those who started full-height backside jumping at the youngest ages (<13 months) and the highest odds for those who started at between 13 and 15 months. Other factors associated with the injury risk in Border Collies were the way in which the dog was acquired (lower odds among those acquired from a rescue or shelter) and the handler age (highest risk for handlers aged 35–44). To aid in interpretation, the results from this same model but with the highest category of ordered categorical variables as the reference are shown in the Supplementary Materials (Table S1 and Supplementary Figure S1).

### 3.3. Prolonged Injury Risk Factors

The final model for factors associated with prolonged injury included two variables in addition to dog age (Table 4). The age at which dogs were reported to have started training with elbow height jumps was associated with prolonged injury, with the highest odds of prolonged injury observed for dogs reported to have started between 13 and 15 months of age and a lower risk among dogs who started between 16–18 or after 18 months of age. Handler medical training was also associated with prolonged injury, with the highest odds observed for the dogs of handlers who were veterinary assistants. A somewhat higher risk was also observed for the dogs of handlers who were in the human healthcare field.

### 3.4. Injury Differences by Height

There was a statistically significant difference in the percentage of dogs reporting any history of digit injury by height, with taller Border Collies much more likely to have a history of a digit injury than shorter ones (Table 5). Although it did not reach the level of statistical significance, taller Border Collies were also more likely to have had a shoulder injury, and shorter Border Collies were more likely to report an injury to the lumbosacral region.

## 4. Discussion

As was hypothesized, Border Collies jumping noticeably above shoulder height had an increased risk of injury. Jumps are the most frequent obstacle on an agility course and could be a source of repetitive stress injuries. Jump height categories are determined based on the height of the dog at the withers and vary based on the overseeing agility organization. Jump height categories are frequently debated as a potential welfare issue and cause of injury, but no definitive correlation has been obtained. Several studies have evaluated the kinematics and kinetics of jumping. One study demonstrated that dogs significantly alter their jump kinematics as the jump height increases, particularly once the height of the jump increases over 125% of the height of the dog at the withers [12]. Another found that the peak vertical force in the forelimbs was higher with higher obstacles [11]. Conversely, a recent study showed no decrease in landing forces when the jump height was decreased from 20 inches to 16 inches [15]. Since these studies primarily used Border Collies, as well as some Border Collie crosses, it is unknown how specific these kinematic and kinetic changes are to Border Collies and how much variation there is between breeds. These changes in kinematics and increased peak vertical forces in dogs jumping above shoulder height could potentially contribute to repetitive stress injuries, particularly in the shoulder, which is the most injured anatomic region [1,11,12]. However, if all breeds have similar changes in kinematics and kinetics during jumping, this may not explain the increase in the injury rate in the Border Collie breed compared to other breeds.

Border Collies jumping noticeably below shoulder height were also more likely to report an injury. This could be due to reverse causality, where the injury resulted in the handler decreasing the jump height after injury recovery. A reduction in agility jump height after injury has been previously reported [16]. It has been hypothesized that reducing the jump height reduces the forces and is therefore safer for dogs after an injury. Based on the study by Pogue et al., this reduction in jump height to reduce the forces does not appear to be valid [15].

It is common practice for handlers to jump young/adolescent dogs at lower jump heights prior to growth plate closure, with the similar thought that it is safer due to decreased forces. However, once again, initial studies in the literature do not necessarily support this [15]. Elbow height jumps have been specifically, but anecdotally, noted as a limit for pre-growth plate closure training. The association between the injury history and the age at starting elbow height jumps may indicate that the repetitive impact of jump training prior to maturity, even at lower heights, has a negative influence on musculoskeletal development, particularly as a higher risk was observed for dogs starting elbow height jumps at younger ages. There was also evidence of a prolonged injury history in dogs starting elbow height jumps at 13–15 months compared to other ages. The reason for this is unknown, but it may be related to the progression of training. It is possible that handlers who start dogs jumping at younger ages limit the number of repetitions or build up training more slowly versus people who wait until their dog is older and push the training progression faster and with a greater intensity, with the goal of starting competition quickly. Why early elbow height jump training increases the injury risk in Border Collies but not agility dogs overall [3] is unknown. It is possible that there are other variables involved, such as the number of jump repetitions, the intensity of the training sessions, or their frequency, that differ between Border Collies training in agility compared to other breeds. Alternatively, the body conformation may play a role in this increased risk; future biomechanical studies would be needed to address this theory. Longitudinal, prospective studies are needed to evaluate various training factors and their relationships with the injury risk over time, particularly training variables in young, developing dogs.

Backside jumping is a technique where the dog is asked to move around to the back side of the jump and take the jump in the opposite direction of the expected line. This requires more deceleration, turning, and collection than when the dog is asked to take the front side of the jump. Among agility trainers and handlers, it is thought that backside jumping is more physically demanding and could lead to injury if performed on a repetitive basis. However, no kinetic or kinematic studies have been performed to look at the differences in physical demands between backside jumping compared to straight-line jumping or jump turns. The injury risk pattern when starting full-height backside jumps was unexpected. The Border Collies that started full-height backside jumps prior to 13 months reported the lowest injury history, but the Border Collies that started at between 13 and 15 months had the highest risk of injury. It is unknown why the injury risk pattern has the distribution reported in these data, and it is unknown why this pattern exists for Border Collies but not for agility dogs overall [3]. It is likely that there are confounding variables that could not be assessed by this survey. Kinetic and kinematic studies of various jumping approaches and techniques are needed to understand the differences in physical demands between these techniques and how they might relate to the injury risk. Prospective studies on the effects of specific exercises and agility training techniques on musculoskeletal development are needed.

The training and competition load, overtraining and fatigue, and their relationship with injury have been studied in human and equine sports [17,18,19,20]. High workloads have been associated with injury and poor performance in both humans and horses [19,20,21,22]. There is no literature evaluating the workload, overtraining, fatigue, and injury in canines. In this context, the finding of an association between the number of trial weekends and injury risk was not unexpected, but the relatively lower risk observed for Border Collies competing for 26 or more weekends per year was unexpected. Workload metrics are complex and studies in human medicine have indicated that single metrics like frequencies or durations are not necessarily indicative of the overall workload or injury risk, which could be the case in this survey, where number of trial weekends does not provide a complete depiction of the total workload [17,22,23]. It is also possible that the retrospective data regarding the number of trial weekends per year are affected by reverse causality, where handlers decrease the number of competitions per year due to a prior dog injury. Alternatively, the decreased risk of injury among these Border Collies competing on 26 or more weekends per year could be due to increased overall fitness. There has been much discussion in human sports performance regarding the ”Training–Injury Prevention Paradox”, which describes the protective nature of an increased training load and corresponding increased injury risk related to workloads that are too low due to decreased fitness [23]. Many studies propose that the best predictor of injury risk is the acute–chronic workload ratio [17,22,23]. The acute workload is the amount of load experienced over a short period of time, often the most recent week, and the chronic workload is the rolling average of the most recent 3–6 weeks of training [23]. It appears that a high chronic workload may be an indication of increased fitness and that the risk of injury is actually attributable to sudden increases, or spikes, in the acute workload [23,24,25]. It is possible that Border Collies that are consistently and frequently competing have a higher chronic workload, indicating a higher level of fitness, and are less likely to have spikes in their acute load. Conversely, dogs that are competing less frequently have a lower level of fitness or they may be at a higher risk of sudden increases in workload if they are not training and competing as consistently. Prospective studies are needed to evaluate the monitoring of canine workload variables and their relationship with injury as this will likely influence agility training and competition planning.

Previous studies have shown correlations between competing in national competitions and an increased injury risk in agility dogs [3,4]. However, the risk pattern for Border Collies is different from that reported in agility dogs overall [3]. Among agility dogs overall, competing at the national level was associated with an increased risk of injury regardless of the number of times that the dog had competed at the national level [3]. However, in Border Collies, the lowest odds of injury were associated with competing in one or > five national competitions, and the highest odds were observed for dogs competing two and particularly three to five times at the national level. It is unknown why this pattern of injury risk in association with the number of national competitions exists in Border Collies but, again, is not seen in the overall agility dog population [3].

Border Collies acquired from rescues or shelters had a reduced risk of injury compared to those acquired from a breeder. It is unknown why dogs of the same breed, acquired in different ways, have different injury risks. It is possible that Border Collies acquired from breeders, particularly those bred for sport, may have personality traits like higher drive and a greater tendency to work through pain or injury, which could predispose them to the development or perpetuation of injuries. Studies on herding dog behavioral genetics and neurochemistry, although not all specific to Border Collies, show that there are distinct genetic variations and neurochemical characteristics that produce the high drive, problem-solving ability, visual orientating, and trainability commonly associated with this breed group [26,27,28,29]. Due to the importance of behavior and its effect on performance in working dogs, such as guide dogs and military working dogs, much research has been performed on selective breeding for desired traits in this population to reduce the attrition rates from working programs. Studies have shown that the traits that determine success are variable based on the type of work that the dog is designated to perform, with confidence, a lack of fear, hyperactivity, and concentration being positive predictors of success in many working dog populations [30,31,32]. It is unknown, however, how these traits correlate with the high rate of musculoskeletal injury that is also seen in this population of dogs [33,34]. It is possible that there are conformational or genetic factors that predispose certain lines of Border Collies to injury, as diseases such as shoulder osteochondrosis, gastrocnemius musculotendinopathy, episodic exercise-induced collapse, and trapped neutrophil syndrome are more common in the breed compared to other breeds [35,36,37]. It is also possible that handlers who acquire Border Collies from breeders are more likely to have higher goals for their dog’s agility career and therefore may be more likely to train with a greater intensity or frequency, which could increase the risk of injury, although no current studies have evaluated this hypothesis. Additional studies would be needed to further elucidate the possible causes of the differences in injury risk between various populations of Border Collies.

The handler age was also correlated with an increased injury risk in Border Collies, with dogs of handlers between 35 and 44 years of age at the highest risk. It is possible that this is a reflection of increased competitiveness in this age group, for which factors of competitiveness have been previously shown to be correlated with an increased risk of injury [3,4]. Other training and competition variables that could not be assessed in this survey could be associated with this age group of Border Collie handlers and could be more directly correlated with the injury risk.

The risk factor associations with prolonged injuries, as defined as injuries keeping dogs out of training for greater than three months, were not as clear. Once again, the age at which dogs were reported to start elbow height jumping was correlated with injury. However, the pattern associated with prolonged injury was different from that of overall injury in Border Collies. The highest risk of prolonged injury was associated with starting elbow height jump training between 13 and 15 months of age, as opposed to overall injury, where the risk was highest in dogs starting this training at <10 months of age. This pattern of injury risk in Border Collies mirrors that of the overall dog population [3]. This is potentially concerning as, depending on the agility organization, dogs may be allowed to compete with full-height jumps at 15 months of age, which would then require training at higher jump heights before starting competition. Handler medical experience was also associated with the risk of prolonged injury, with a particularly higher risk observed in the dogs of handlers who were veterinary assistants and a somewhat higher risk for the dogs of handlers who were in the human healthcare field. It is unknown why these two particular demographic groups would have a higher risk of prolonged injury in their dogs. Handlers in the medical professions may be more cautious in returning their dog to agility training after injury and may be more likely to keep their dogs out of agility longer than other handlers for similar injuries, thereby skewing the data.

Despite being a single breed, Border Collies can vary dramatically in height as well as structure. While a retrospective survey methodology cannot evaluate for structure and its relationship with injury, the statistical analysis did suggest that there may be different injury risks based on the height of the Border Collie. Taller Border Collies were more likely to incur digit injuries. Contact obstacles, particularly the A-frame, and weave pole bases have been hypothesized to be a potential source of digit injuries [38]. It is possible that the longer lever arm in taller dogs increases the forces through the terminal segment (i.e., the digits), particularly during deceleration on contact obstacles and during lateral movements such as weave pole performance and turning. Although it did not reach statistical significance, there was a trend towards an increased risk of shoulder injuries in taller Border Collies. This could be due to the increased external moments on the shoulder joint in taller dogs, thereby increasing the muscular forces required to counteract the external moments. The increase in muscle engagement could result in increased muscle and tendon injuries due to fatigue, overuse, and repetitive stress. In shorter Border Collies, there was a trend towards an increased risk of lumbosacral injury. Depending on the agility organization, shorter Border Collies may be jumping noticeably above shoulder height, and jumping above shoulder height was associated with a greater general injury risk, as discussed previously. However, the increased risk of injury specifically to the lumbosacral region may be related to the increased degree of lumbosacral extension required for them to jump the same height jump as their taller counterparts. It is currently thought that an increased or altered mechanical load on the lumbosacral disc contributes to the high prevalence of lumbosacral disc degeneration [39]. It is possible that the increased degree of extension from jumping relatively higher jump heights could increase the mechanical load on the lumbosacral disc and increase the risk of pathology at this site.

There are many variables that could be associated with the injury risk in Border Collies that could not be evaluated in a retrospective owner-reported survey. It has been hypothesized that an increased injury risk in canine agility is directly related to increased speed, as has been demonstrated in human athletes, racehorses, and racing greyhounds [40,41,42]. It is also purported that Border Collies, as a breed, are faster than other breeds during agility performance. However, to the authors’ knowledge, this is not supported by any published, peer-reviewed formal analysis. It is possible, if the speeds of Border Collies are higher than other breeds as they participate in agility, that the increased speed could increase their injury risk over other breeds. Other breed-specific factors that could not be evaluated but might contribute to the injury risk include genetic factors, such as those related to developmental orthopedic disease. Structure and conformation can affect kinematics and kinetics so that biomechanics vary by breed and even within breeds that have substantial conformational variety [43,44,45,46,47]. With regard to Border Collies, it has been specifically shown that the kinetics at a walk and trot differ between Border Collies and Labrador Retrievers [45]. It is likely that the conformation and structure also affect the kinematics and kinetics during agility performance, although how this is related to the injury risk is unknown.

By design, retrospective owner-based surveys have inherent limitations, including possible self-selection bias and participant recall. The selected cutoff of 3 months for the prolonged injury category was somewhat arbitrary. However, previous studies have shown that, for more severe shoulder tendinopathies and after tibial plateau leveling osteotomy surgery, many dogs require 4 to 6 months to return to sport [48,49,50]. Therefore, an injury that keeps a dog out of training for more than 3 months is likely to be more severe than those injuries where the dog can return within a 3-month timeframe, although there are many other variables that could potentially influence the timeframe of return to sport that cannot be captured in a retrospective survey. This survey also only asked about training and competition variables at the time of the survey’s completion. It did not ask about training and competition practices prior to the injury or how the injury affected the current training and competition practices. It is highly likely that a history of injury influenced some of the reported variables, and this could not be evaluated.

## 5. Conclusions

Seven risk factors were identified as correlated with the injury risk in Border Collies competing in agility competitions. As hypothesized, Border Collies jumping above shoulder height had an increased risk of injury, possibly due to changes in kinematics and kinetics. However, those currently jumping below shoulder height also had an observed increase in risk, potentially due to handlers reducing the jump height due to the prior injury history. Competing at the national level was associated with the injury risk, but in a pattern largely counter to our hypothesis. The observed association between the risk and the age at starting elbow height jumps may indicate that the repetitive impact of jumping prior to maturity negatively influences musculoskeletal development, thereby increasing the risk of future injury.

These data provide valuable information about the possible risk factors for injury in Border Collies, a breed overrepresented in agility competition. Additionally, these data provide guidance for further prospective studies and indicate a need for studies evaluating the biomechanics of jumping and the effect of musculoskeletal development and their relationships with injury. There is also an apparent need for studies evaluating the effect of workload metrics on the development of injuries in agility dogs. Because some of the patterns of injury risk were different for Border Collies compared to agility dogs overall, additional studies on Border Collies’ conformation, structure, behavior, and genetics, and their relationship with injury, are indicated.

## Figures and Tables

**Table 1:** Reported injury history among Border Collies and non-Border Collies in the sample. The column percentages are given both as a percentage of all dogs in the sample (“all dogs”) and among dogs with any reported injury history.

	All Dogs		Dogs with Injury History	
	Border Collies (*n* = 934)	All Others (*n* = 3263)	Border Collies (*n* = 488)	All Others (*n* = 1251)
Any injury (>1 week)	488 (52.3%)	1251 (38.3%)	488 (100%)	1251 (100%)
Any prolonged injury (>3 months)	195 (20.9%)	434 (13.3%)	195 (40.0%)	434 (34.7%)
Number of reported injuries *				
One	235 (25.2%)	635 (19.5%)	235 (48.5%)	635 (51.0%)
Two	138 (14.8%)	384 (11.8%)	138 (28.5%)	384 (30.8%)
Three	61 (6.6%)	140 (4.3%)	61 (12.6%)	140 (11.2%)
Four	21 (2.3%)	40 (1.2%)	21 (4.3%)	40 (3.2%)
Five or more	30 (3.2%)	47 (1.4%)	30 (6.2%)	47 (3.8%)

* Data available on 931 Border Collies and 3258 other dogs.

**Table 2:** All factors considered in model building. Variables moved from step 1 to step 2 if they were significant (p < 0.2) in models adjusted for the age of the dog. In step 2, backward selection was conducted within each block of variables until all variables in the block were significant (p < 0.2). Final model building (step 3) was conducted via backward selection starting with all remaining variables after the model building in step 2.

	*p* < 0.2 in Age-Adjusted Models (Step 1)	*p* < 0.2 in Block Model Building (Step 2)	Retained in Final Model (Step 3)
**Demographic Factors**			
Height and weight together	✓		
Country/region	✓		
Age when bringing dog home	✓		
How acquired (breeder, rescue, other)	✓	✓	✓
Acquired with agility in mind			
Agility main sport focus			
Sex/neuter status			
Front dew claws			
Rear dew claws			
Growth plate X-rays performed			
Handler current age	✓	✓	✓
Handler gender			
Handler education			
Handler profession			
Handler medical training	✓		
Handler agility experience			
Handler competed at national level			
Handler competed at international level	✓		
**Competition Factors**			
Primary organization	✓		
Dog’s highest level achieved			
Jump height relative to dog height	✓	✓	✓
Approach to competition planning	✓		
Advance competition planning			
Trial weekends per year	✓	✓	✓
Average runs per trial day	✓		
Average days per trial weekend			
Grass surface	✓		
Dirt surface	✓		
Sand surface			
Artificial turf surface			
Foam surface	✓	✓	
Rubber mat surface	✓	✓	
Other surface			
Number of times that this dog has competed at a national competition	✓	✓	✓
Number of times that this dog has competed at an international competition			
**Training Factors**			
Age when starting any agility training	✓	✓	
Age at first fun match			
Age at first trial			
Age when starting any jump training			
Age when starting elbow height jumps	✓	✓	✓
Age when starting full height jumps			
Age when starting backside jump training	✓		
Age when starting backside at full height	✓	✓	✓
Age when starting any tunnel training			
Age when starting curved tunnel training			
Age when starting A-frame training			
Age when starting dogwalk training	✓	✓	
Age when starting teeter training			
Age when starting any weave training	✓	✓	
Age when starting sequencing with closed weaves			
A-frame contact behavior			
Dogwalk contact behavior			
Teeter contact behavior	✓		
Weave training method			

**Table 3:** Coefficients from final adjusted model of risk factors of any injury.

	Adjusted OR (95% CI)	Adjusted *p*-Value	N (%) *
Dog age (per 1 year older)	1.20 (1.12, 1.29)	<0.001	
How acquired		0.021	
Breeder	REFERENCE		630 (79.3)
Rescue/Shelter	0.50 (0.30, 0.82)		101 (12.7)
Other	0.99 (0.56, 1.76)		64 (8.0)
Handler current age		0.019	
18–24	REFERENCE		44 (5.5)
25–34	1.35 (0.63, 2.89)		114 (14.3)
35–44	2.33 (1.09, 5.00)		127 (16.0)
45–54	0.99 (0.48, 2.08)		177 (22.3)
55–64	1.12 (0.54, 2.31)		231 (29.1)
65+	0.94 (0.42, 2.08)		102 (12.8)
Jump height difference		0.001	
Jumping >4” below height	REFERENCE		75 (9.4)
Jumping 2–4” below height	0.45 (0.21, 0.96)		162 (20.4)
Jumping 0–2” below height	0.45 (0.23, 0.89)		254 (32.0)
Jumping 0–2” above height	0.33 (0.17, 0.64)		158 (19.9)
Jumping 2–4” above height	0.53 (0.26, 1.07)		80 (10.1)
Jumping >4” above height	0.95 (0.43, 2.13)		66 (8.3)
Trial weekends per year		0.043	
<5 weekends	REFERENCE		57 (7.1)
5–10 weekends	2.08 (1.04, 4.16)		145 (18.2)
11–15 weekends	2.23 (1.15, 4.33)		232 (29.2)
16–20 weekends	2.35 (1.18, 4.66)		195 (24.5)
21–25 weekends	3.12 (1.49, 6.52)		108 (13.6)
26+ weekends	1.44 (0.62, 3.34)		58 (7.3)
Times competed at national level		0.004	
0 (never)	REFERENCE		416 (52.3)
1	0.58 (0.36, 0.93)		110 (13.8)
2	1.16 (0.63, 2.14)		65 (8.2)
3–5	1.83 (1.05, 3.18)		95 (12.0)
>5	0.70 (0.41, 1.21)		109 (13.7)
Age elbow height jumps		0.026	
<10 months	REFERENCE		95 (12.0)
10–12 months	0.59 (0.32, 1.09)		76 (9.6)
13–15 months	0.93 (0.51, 1.70)		196 (24.7)
16–18 months	0.48 (0.23, 0.98)		319 (40.1)
>18 months	0.55 (0.26, 1.16)		109 (13.7)
Age backside at full height		0.010	
<13 months	REFERENCE		417 (52.5)
13–15 months	2.96 (1.18, 7.43)		29 (3.7)
16–18 months	1.39 (0.56, 3.50)		137 (17.2)
> 18 months	1.90 (0.77, 4.68)		212 (26.7)

* 795 observations in the final model.

**Table 4:** Coefficients from final adjusted model of risk factors of prolonged injury.

	Adjusted OR (95% CI)	Adjusted *p*-Value	N (%) *
Dog age (per 1 year older)	1.26 (1.18, 1.33)	<0.001	
Handler medical training/experience		0.030	
None of these	REFERENCE		668 (75.7)
Veterinarian	0.77 (0.30, 1.95)		37 (4.2)
Licensed vet tech	0.90 (0.34, 2.38)		28 (3.2)
Veterinary assistant	3.11 (1.38, 7.02)		29 (3.3)
Human healthcare professional	1.56 (0.97, 2.50)		120 (13.6)
Age at elbow height jumps		0.012	
<10 months	REFERENCE		113 (12.8)
10–12 months	0.97 (0.47, 2.01)		81 (9.2)
13–15 months	1.65 (0.84, 3.24)		215 (24.4)
16–18 months	0.81 (0.35, 1.84)		356 (40.4)
>18 months	0.77 (0.34, 1.73)		117 (13.3)

* 882 observations in the final model.

**Table 5:** Percentage of injured dogs reporting an injury to a specific location by height category.

Injury Location	<19 Inches (*n* = 203)	19–21 Inches (*n* = 529)	>21 Inches (*n* = 172)	*p* for Difference
Shoulder	27 (13.3%)	81 (15.3%)	35 (20.4%)	0.16
Iliopsoas	21 (10.3%)	67 (12.7%)	26 (15.1%)	0.32
Digits (toes)	11 (5.4%)	66 (12.5%)	25 (14.5%)	0.008 ^
Lumbar spine/lumbosacral	19 (9.4%)	50 (9.5%)	7 (4.1%)	0.075
Stifle	15 (7.4%)	29 (5.5%)	14 (8.1%)	0.38
Paw pad	7 (3.5%)	35 (6.6%)	14 (8.1%)	0.14
Any other location	9 (4.4%)	27 (5.1%)	9 (5.2%)	n/a

^ significant at 0.05 level after Holm correction for multiple comparisons (among 6 body parts with more than 5% of dogs reporting injury to that location).

## Data Availability

The data presented in this study are available on request from the corresponding author.

## Supplementary Materials

The following supporting information can be downloaded at: https://www.mdpi.com/article/10.3390/ani14142081/s1, Figure S1. Coefficients from final adjusted model of risk factors of any injury using highest category as the reference; Table S1. Coefficients from final adjusted model of risk factors of any injury using highest category as the reference.
