# Supplementary material for: Risk Factors for Injury in Border Collies Competing in Agility Competitions
Source: Animals (Basel). 2024 Jul 16;14(14):2081. doi: 10.3390/ani14142081 (PMC11273924; doi:10.3390/ani14142081)
Supplement: Supplementary file 1 [file animals-14-02081-s001.zip › animals-3060047-supplementary.pdf]

Supplementary Materials

Table S1. Coefficients from final adjusted model of risk factors of any injury using highest category as the reference.

	Adjusted OR (95% CI)	Adjusted *p*-value	N (%)*
Dog age (per 1 year older)	1.20 (1.12, 1.29)	<0.001	
How acquired		0.021	
Breeder	1.01 (0.57, 1.80)		630 (79.3)
Rescue / Shelter	0.50 (0.25, 1.01)		101 (12.7)
Other	REFERENCE		64 (8.0)
Handler current age		0.019	
18-24	1.07 (0.48, 2.37)		44 (5.5)
25-34	1.44 (0.78, 2.66)		114 (14.3)
35-44	2.49 (1.37, 4.53)		127 (16.0)
45-54	1.06 (0.62, 1.82)		177 (22.3)
55-64	1.20 (0.72, 1.99)		231 (29.1)
65+	REFERENCE		102 (12.8)
Jump height difference		0.001	
Jumping >4″ below height	1.05 (0.47, 2.35)		66 (8.3)
Jumping 2-4″ below height	0.47 (0.23, 0.98)		80 (10.1)
Jumping 0-2″ below height	0.47 (0.25, 0.89)		158 (19.9)
Jumping 0-2″ above height	0.34 (0.19, 0.62)		254 (32.0)
Jumping 2-4″ above height	0.56 (0.30, 1.04)		162 (20.4)
Jumping >4″ above height	REFERENCE		75 (9.4)
Trial weekends per year		0.043	
<5 weekends	0.69 (0.30, 1.61)		57 (7.1)
5-10 weekends	1.44 (0.72, 2.89)		145 (18.2)
11-15 weekends	1.55 (0.81, 2.95)		232 (29.2)
16-20 weekends	1.63 (0.84, 3.16)		195 (24.5)
21-25 weekends	2.17 (1.06, 4.42)		108 (13.6)
26+ weekends	REFERENCE		58 (7.3)
Times competed at National level		0.004	
0 (never)	1.42 (0.83, 2.44)		416 (52.3)
1	0.82 (0.44, 1.53)		110 (13.8)
2	1.66 (0.82, 3.33)		65 (8.2)
3-5	2.61 (1.37, 4.96)		95 (12.0)
>5	REFERENCE		109 (13.7)
Age elbow height jumps		0.026	
< 10 months	1.82 (0.86, 3.83)		76 (9.6)
10-12 months	1.08 (0.59, 1.96)		196 (24.7)
13-15 months	1.69 (0.97, 2.96)		319 (40.1)
16-18 months	0.87 (0.46, 1.65)		109 (13.7)
> 18 months	REFERENCE		95 (12.0)
Age backside at full height		0.010	
< 13 months	0.53 (0.21, 1.30)		29 (3.7)
13-15 months	1.56 (0.96, 2.53)		137 (17.2)
16-18 months	0.73 (0.49, 1.09)		212 (26.7)
> 18 months	REFERENCE		417 (52.5)

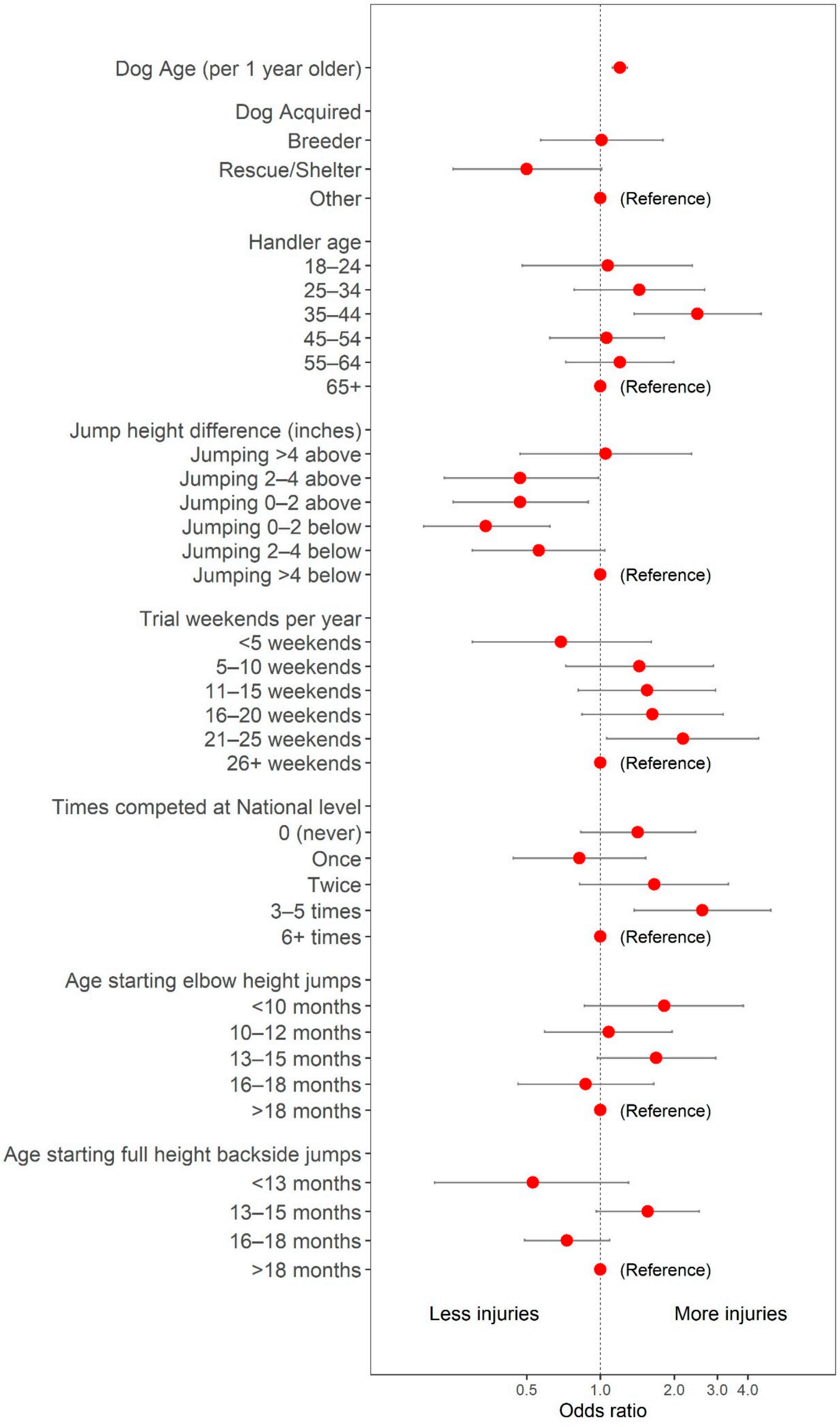

Figure S1. Coefficients from final adjusted model of risk factors of any injury using highest category as the reference.
